# Association between carotid artery hemodynamics and neurovascular coupling in cerebral small vessel disease: an exploratory study

**DOI:** 10.3389/fnagi.2025.1536552

**Published:** 2025-02-07

**Authors:** Peng Zeng, Bang Zeng, Xiaohua Wang, Feiyue Yin, Binglan Li, Lisha Nie, Lin Tian, Dan Luo, Yongmei Li

**Affiliations:** ^1^Department of Radiology, First Affiliated Hospital of Chongqing Medical University, Chongqing, China; ^2^MRI Research, GE Healthcare (China), Beijing, China; ^3^Circle Cardiovascular Imaging, CVI Clinical Application China, Shanghai, China

**Keywords:** 4D flow MRI, hemodynamics, resting-state functional MRI, multi-delay arterial spin labeling, neurovascular coupling, cerebral small vessel disease

## Abstract

**Background:**

Recent studies have linked disrupted cerebral hemodynamics, including pulsatility index (PI) and wall shear stress (WSS), with neuroimaging features of cerebral small vessel disease (CSVD). Cerebral neurovascular coupling (NVC) dysfunction is an important pathophysiological mechanism of CSVD. However, evidence linking the features of carotid artery hemodynamics to cerebral NVC is still lacking.

**Objective:**

This study is aimed to explore the impact of PI and WSS on NVC and cognitive performance in CSVD patients using neuroimaging.

**Methods:**

This study included 52 CSVD patients and 41 healthy controls. Carotid artery PI and WSS were measured using 4D flow magnetic resonance imaging (MRI). NVC was assessed through voxel-wise correlations between cerebral blood flow and the amplitude of low-frequency fluctuations. Multiple linear regression was used to investigate correlations between them.

**Results:**

CSVD patients showed elevated PI in the C2 and C4 segments of the internal carotid artery and reduced WSS in the common carotid artery compared to controls. NVC measurements were significantly diminished in CSVD patients. Multiple linear regression analysis indicated significant correlations between reduced WSS and impaired NVC as well as between reduced PI and impaired NVC, but not between PI, WSS, and cognitive scores.

**Conclusion:**

Reduced WSS and PI in CSVD patients are associated with impaired NVC. These findings provide insights into the mechanisms underlying CSVD and suggest that hemodynamic abnormalities may serve as indicators of neurovascular dysfunction in early-stage CSVD.

## 1 Background

Cerebral small vessel disease (CSVD) primarily affects a class of small vessels in the brain characterized by white matter hyperintensities (WMH), lacunar infarcts (LI), enlarged perivascular spaces (EPVS) in basal ganglia (BG) and centrum semiovale (CS), and cerebral microbleeds (CMB) in neuroimaging (Ter Telgte et al., 2018).

Recent studies mainly focused on the associations between hemodynamic parameters and CSVD neuroimaging markers, particularly the relationships between pulsatility index (PI) and wall shear stress (WSS) with WMH, atrophy, LI, and CMB (Poels et al., 2012; Jochemsen et al., 2015; Mitchell et al., 2011; Wåhlin and Nyberg, 2019; Birnefeld et al., 2020; Pahlavian et al., 2021; Webb et al., 2012; Wåhlin et al., 2014; Liu et al., 2016). Elevated arterial PI, indicative of increased vessel stiffness, is often attributed to vascular risk factors and impaired vasodilation (Wardlaw et al., 2019). It has been hypothesized that the stiffened vessels would be less capable to dampen pressure and pulsatility, leading to more pulsatile energy from upstream dissipating in the brain tissue (Mitchell, 2015). This contributes to endothelial dysfunction, blood–brain-barrier impairment (Wåhlin and Nyberg, 2019; de Montgolfier et al., 2019; Garcia-Polite et al., 2017), and consequently disruption of the neurovascular unit (Hawkins and Davis, 2005). Beyond relationships between abnormal PI and structural disruptions, one study also demonstrated a significant correlation between PI and hippocampal perfusion (Vikner et al., 2021). Conversely, decreased WSS correlates with endothelial changes and vascular remodeling (Soulat et al., 2020). These alterations combine with systemic risk factors to further contribute to atherosclerotic plaque formation, in turn exacerbating flow disruption and promoting growth of the fibroinflammatory lipid plaque (Cunningham and Gotlieb, 2005). Besides, decreased WSS was previously proposed as a risk factor for CSVD in the common carotid artery (Liu et al., 2016). In all, hemodynamic abnormality plays an essential role in the pathophysiology of CSVD, especially in structural integrity.

Regional cerebral blood flow (CBF), which is associated with glucose oxidative metabolism, is correlated with spontaneous neural activation. This mechanism is known as neurovascular coupling (NVC), and is crucial for maintaining homeostasis in the cerebral microenvironment. The combination of cerebral blood flow (CBF) and functional measures to represent the integrity of neurovascular coupling is strongly associated, both at rest and in response to tasks (Liang et al., 2013). On the other hand, previous studies have demonstrated that alterations in CBF and amplitude of low-frequency fluctuation (ALFF) were related to CSVD, respectively (Wang et al., 2021; Pahlavian et al., 2021). The neurovascular uncoupling, combining CBF and functional measurements (Liang et al., 2013; Li et al., 2024), was related to deterioration of neuroimaging markers, including WMH, CMB, LI, and cerebrovascular endothelial dysfunction in CSVD (Mitchell, 2015). All these investigations suggest that NVC dysfunction is closely correlated with both structural integrity and functional normality in CSVD.

These findings showed correlations between abnormal hemodynamics and impaired parenchyma as well as between neurovascular dysfunction and cerebral disruption, suggesting a potential relationship between hemodynamics and NVC in CSVD. However, the direct impact of abnormal hemodynamics on neurovascular dysfunction in CSVD remains insufficiently investigated. Additionally, there have been few attempts to combine multiple hemodynamic measurements to investigate alterations in CSVD.

PI and WSS were derived from 4D phase-contrast MRI (4D flow) in our study, which simultaneously images a wide range of blood vessels while maintaining accuracy with three-directional velocity encoding and has been developed as an effective method for visualization and quantification of blood hemodynamics. Additionally, cerebral blood flow (CBF) is derived from multi-delay arterial spin labeling (ASL) and acquired for a more accurate assessment of regional perfusion compared to traditional ASL with fixed post-labeling (Buxton et al., 1998), especially in CSVD patients accompanied by universal vascular impairment (Jezzard et al., 2018). Consequently, combining carotid artery hemodynamics and cerebral NVC may provide us with more direct and novel pathophysiological mechanisms underlying CSVD.

This study estimated the PI and WSS in the carotid arteries using 4D flow MRI and NVC measurements in the brain using blood oxygen level dependent (BOLD) and multi-delay ASL and further explored the relationships among PI, WSS, NVC measurements, and cognitive scales in CSVD patients. We hypothesized that distinct interactions between proximal hemodynamics and distal NVC may be evident.

## 2 Materials and methods

### 2.1 Participants

The study was performed in accordance with the latest version of the Declaration of Helsinki and approved by the ethics committee of the First Affiliated Hospital of Chongqing Medical University. Written informed consent was obtained from each participant. A total of 52 CSVD patients and 41 healthy controls (HC) were recruited between February 2023 and July 2024, with mean ages of 53.46 (5.42) and 59.67 (7.15), respectively. The inclusion criteria were as follows: (1) age > 45 years, (2) right-handed, and (3) tolerating a 1 h MRI scan. The exclusion criteria were as follows: (1) a history of head trauma; (2) disorders related to psychiatry and other neurological or systemic comorbidities; (3) structural brain lesions, including severe stroke, vascular malformation, moyamoya, tumor, and intracranial infection; and (4) MRI contraindications, artifacts, and images with low quality.

### 2.2 Clinical data

The baseline demographics were collected via face-to-face interviews by well-trained researchers, including age, sex, educational attainment, stroke history, medical history (hypertension, diabetes, hypercholesterolemia, hyperhomocysteinemia, atrial fibrillation, and coronary artery disease), smoking, and drinking status. Vascular risk factors (VRF) were quantified using a total score, which was assigned one point for each positive medical history, smoking, and alcohol consumption.

Global cognition status was identified using the Mini-Mental State Examination (MMSE) (Folstein et al., 1975) and Montreal Cognitive Assessment (MoCA) (Nasreddine et al., 2005), jointly for a more comprehensive evaluation of cognition, both adding one score to correct education bias when education years were below 12.

### 2.3 MRI acquisition

All participants underwent a 3.0-tesla MRI scan (Signa Premier, GE Medical Systems, USA) equipped with a commercial 48-channel head coil. BOLD, multi-delay ASL, 3D T1-weighted imaging using magnetization-prepared rapid acquisition with gradient echo (T1-MPRAGE), magnetic resonance imaging compilation (MAGiC) (Tanenbaum et al., 2017), susceptibility-weighted imaging (SWI), and 4D phase-contrast MRI (4D flow MRI) were performed. Specifically, the parameters of 4D flow MRI were as follows: slice thickness 2.0 mm, no gaps, FOV 360 × 324 mm^2^, matrix size 192 × 192, reconstructed voxel size 1.41 × 1.41 × 2 mm^3^, number of slices 82, and velocity encoding 80 cm/s. Detailed parameters for the rest sequences were in Supplementary material.

### 2.4 CSVD-total-burden-score evaluation

CSVD-total-burden-score was estimated using Rothwell’s criteria (score: 0–6) (Lau et al., 2017), according to which subjects with score 0 were grouped into HC while subjects above score 0 were grouped into CSVD. We additionally used Wardlaw’s criteria (scored 0–4) for all subjects for a more comprehensive evaluation of CSVD-total-burden (Wardlaw et al., 2013). Specifically, both criteria consisted of WMH, CMB, LI, and EPVS. The intraclass correlation coefficient (ICC) was used to evaluate interobserver reliability (Koo and Li, 2016). Further details of evaluation are provided in the Supplementary material.

### 2.5 NVC measurements from BOLD and ASL

**Figure 1:**
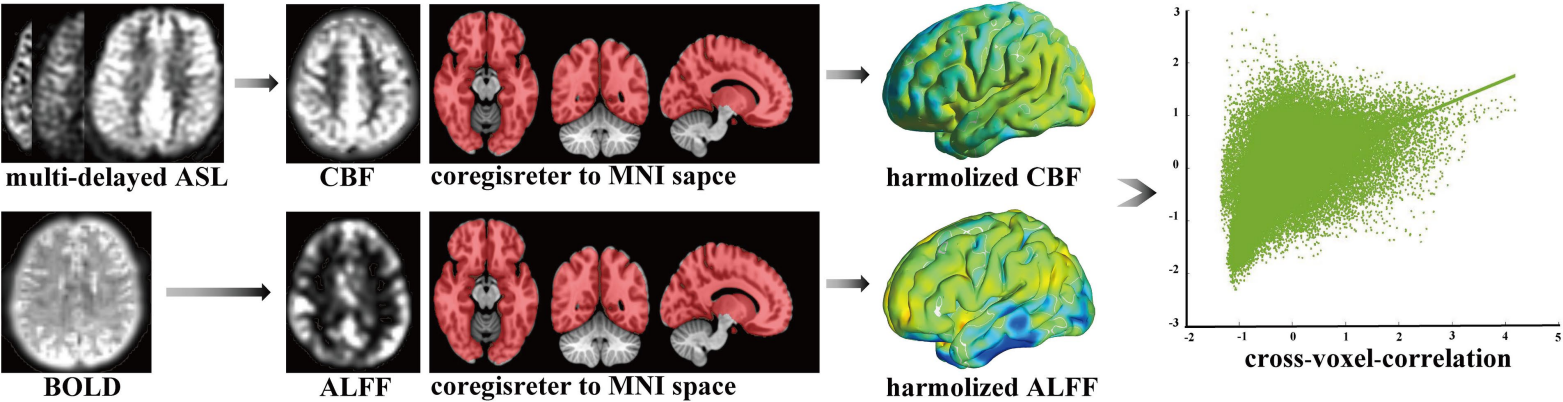
Calculation of cross-voxel-correlation. ASL, arterial spin labeling; BOLD, blood oxygen level dependent; CBF, cerebral blood flow; ALFF, amplitude of low-frequency fluctuation; MNI, Montreal Neurological Institute space.

Bold was processed to obtain the ALFF, including removing the first 10 time points, slice timing, realignment, spatial normalization, detrending, regression of nuisance covariates, filtering, z-scoring, and smoothing. ASL-derived CBF was processed using spatial normalization, z-scoring, and smoothing. Using the above ALFF and CBF, the cross-voxel-correlation coefficient between them was calculated for NVC measurements across all voxels through the global cerebrum for each individual. We also removed the cerebellum because of the limited scanning scale, as shown in Figure 1. The higher cross-voxel-correlation coefficient corresponded to stronger NVC, as applied previously (Roberts et al., 2023). Furthermore, the regional homogeneity (ReHo) was also calculated as another cross-voxel-correlation coefficient to demonstrate the robustness of the results. Detailed processes are provided in the Supplementary material.

### 2.6 Hemodynamic measurements from 4D flow

**Figure 2:**
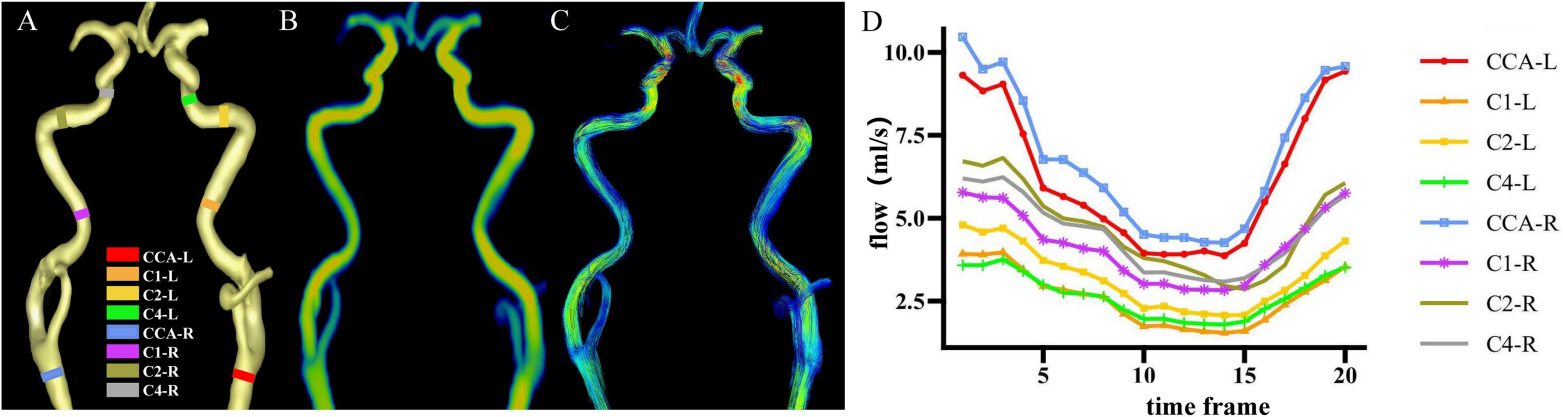
Cross-sections of hemodynamic measurements. **(A)** Eight vessel segments placed with planes perpendicular to the vessel orientations from a representative individual. **(B)** Corresponding flow rate mapping. **(C)** Corresponding path-line mapping. **(D)** Pulsatile flow waveforms of eight vessel segments through the cardiac cycle from a representative individual. CCA, common carotid artery; C1, cervical segment of internal carotid artery; C2, petrous segment of internal carotid artery; C4, cavernous segment of internal carotid artery; R, right; L, left.

Cvi42 software^1^ was used for 4D flow post-processing as previously applied to the cranial artery (Xie et al., 2024). The steps followed were as follows: (1) applying offset correction and phase anti-aliasing for original mapping under the small vessel model; (2) setting the start and end points manually on both sides of the carotid artery for automatic centerline tracking; and (3) placing planes of interest along vessels and adjusting them perpendicular to the centerline of vessels both manually and automatically. To exclude the hemodynamic effect of the physiologic vascular tortuosity, four arterial segments running straight were selected in both the left and right sides of the common carotid artery (CCA), the cervical segment of the internal carotid artery (C1), the petrous segment of the internal carotid artery (C2), and the cavernous segment of the internal carotid artery (C4), based on the Bouthillier standard commonly used in clinical practice (Bouthillier et al., 1996), after which the contour line of each vessel segment was produced automatically; (4) checking and adjusting to make the contour line and real vascular edge overlay completely for each frame across the cardiac cycle and for all vessel segments; (5) extracting data from pulsatile vessel waveforms; and (6) calculating the values of PI-area, PI-rate, and WSS, which represent arterial pulsatility in area, arterial pulsatility in flow, and wall shear stress, respectively. Specific calculations applied were as follows: 1. PI-area = (Amax − Amin)/Amean. (A: cross-sectional area mm^2^). 2. PI-rate = (Qmax − Qmin)/Qmean (Q: flow rate, mL/s). 3. WSS = WSSmean (Pa). The max, min, and mean values were defined as the maximum, minimum, and mean values, respectively, within one cardiac cycle. The values of the bilateral carotid artery were averaged for PI-area, PI-rate, and WSS (Xie et al., 2024). Because small lumen size in intracranial arteries was presented with a low signal-to-noise ratio and serrated edge. We constrained measurements in the common carotid artery (CCA) and C1, C2, and C4 segments of the internal carotid artery, as shown in Figure 2.

### 2.7 Statistical analysis

Continuous variables were presented as mean (SD) for normally distributed data and medians (interquartile range) for non-normally distributed data, whereas the categorical variables were presented as counts (percentage). The variables’ normality was assessed using the Shapiro–Wilk test due to the small sample size.

The intergroup differences in quantitative variables were analyzed using an independent two-sample t-test for parametric data and the Mann–Whitney U test for non-parametric data, and categorical variables were analyzed using the Chi-squared (*χ*^2^) test. Intergroup comparisons of PI-area, PI-rate, and WSS were adjusted forage (Roberts et al., 2023; Eigenbrodt et al., 2006; Irace et al., 2012) using analysis of covariance (ANCOVA) for normally distributed data and ANCOVA after aligned rank transformation for non-normally distributed data.

Multiple linear regression in the CSVD group was performed to investigate the relationship between PI-area, PI-rate, WSS, cognitive scales (MMSE and MoCA), and NVC measurements. The NVC measurement and cognition scale score were the dependent variables, whereas the PI-area, PI-rate, and WSS in all vessel segments were the independent variables. In the analyses, Model 1 controlled for nothing, Model 2 controlled for age, sex, and education, and Model 3 controlled for age, sex, education, VRF-total-score, CS-EPVS, BG-EPVS, CMB, LI, and WMH Fazekas scores. In all regression analyses, a standardized beta coefficient was used, and a variable inflation factor (<5) was used to avoid multicollinearity issues.

Ordinal logistic regression in the CSVD group was used to investigate the association between PI-area, PI-rate, WSS, and CSVD-total-burden-score based on both Rothwell’s and Wardlaw’s criteria. The CSVD-total-burden-score was the dependent variable, whereas PI-area, PI-rate, and WSS were the independent variables. In the analysis, Model 1 controlled for nothing, and Model 2 controlled for age, sex, education, and VRF-total-score. Results were presented as odds ratios (OR) with 95% confidence intervals (95% CI).

Statistical Package for the Social Sciences (SPSS) software (version 25.0, IBM SPSS Inc., Chicago, IL, USA) was used for statistical analysis. *p* < 0.05 was defined as statistically significant.

## 3 Results

### 3.1 Demographics and cognition assessments

Our study included 41 HC and 52 CSVD patients. Compared to HC, patients with CSVD were significantly older and had an increased incidence of hypertension (*T* = −4.61, *p* < 0.01; *χ*^2^ = 4.68, *p* = 0.03). CSVD patients tended to have lower scores on the MoCA, but the difference was not statistically significant (*U* = 870, *p* = 0.13). No significant differences were found in the other demographic indicators. The details are shown in Table 1.

**Table 1:** Demographics, cognition scale, CSVD-total-burden, NVC measurement, and hemodynamic measurement.

	HC (*n* = 41)	CSVD (*n* = 52)	*T*/*χ*^2^/*U*/*F*	*ρ*
Demographics				
Age	53.46 (5.42)	59.67 (7.15)	−4.61	<0.001 ***
Education	12.00 (9.00–15.00)	12.00 (9.00–15.00)	936.5	0.31
Gender (F)	25 (61%)	22 (42%)	3.2	0.07
Hypertension (Y)	10 (24%)	24 (46%)	4.68	0.030*
Hyperlipoidemia (Y)	14 (34%)	13 (25%)	0.93	0.34
Coronary disease (Y)	0 (0%)	0 (0%)	N/A	N/A
Diabetes mellitus (Y)	6 (15%)	11 (21%)	0.65	0.42
Atrial fibrillation (Y)	0 (0%)	0 (0%)	N/A	N/A
Hyperhomocysteinemia (Y)	0 (0%)	0 (0%)	N/A	N/A
Alcohol (Y)	7 (17%)	11 (21%)	0.25	0.62
Smoke (Y)	6 (15%)	14 (27%)	2.05	0.15
VRF-total-score	1.00 (1.00–2.00)	1.00 (1.00–2.00)	922	0.25
Cognition scale				
MMSE	29.00 (28.00–30.00)	29.00 (28.00–30.00)	880.5	0.14
MoCA	26.00 (24.00–27.00)	24.82 (22.00–27.75)	870	0.13
CSVD-total-burden				
Rothwell’s criteria (0–6)	0 (0–0)	1 (1–2)	N/A	N/A
Wardlaw’s criteria (0–4)	0 (0–0)	1 (1–2)	N/A	N/A
NVC measurement				
NVC (GM)	0.28 (0.09)	0.22 (0.08)	7.4	0.008 **
NVC (global cerebrum)	0.47 (0.06)	0.39 (0.09)	9.53	0.003 **
Hemodynamic measurement				
PI-rate-CCA	0.94 (0.13)	0.96 (0.2)	0.76	0.386
PI-rate-C1	0.66 (0.12)	0.7 (0.16)	2.89	0.092
PI-rate-C2	0.7 (0.12)	0.71 (0.16)	7.66	0.007 **
PI-rate-C4	0.59 (0.14)	0.62 (0.14)	5.3	0.024 *
PI-area-CCA	0.38 (0.12)	0.34 (0.13)	1.56	0.215
PI-area-C1	0.26 (0.09)	0.28 (0.09)	0.29	0.592
PI-area-C2	0.26 (0.08)	0.27 (0.10)	0.92	0.34
PI-area-C4	0.21 (0.10)	0.22 (0.09)	1.93	0.169
WSS-CCA	0.27 (0.06)	0.23 (0.06)	15.75	<0.001***
WSS-C1	0.38 (0.07)	0.37 (0.09)	0.45	0.504
WSS-C2	0.36 (0.05)	0.36 (0.07)	0.19	0.66
WSS-C4	0.41 (0.09)	0.39 (0.09)	0.76	0.39

The quantitative variable was presented as mean (SD) for normal distribution and median (interquartile) for abnormal distribution; the categorical variable was presented as number (percentage). HC, healthy control; CSVD, cerebral small vessel disease; NVC, neurovascular coupling; MMSE, Mini-Mental State Examination; MoCA, Montreal Cognitive Assessment; GM, grey matter; VRF, vascular risk factor; M, male; F, female; Y, yes; N/A, not applicable; PI, pulsatility index; WSS, wall shear stress; CCA, common carotid artery; C1, cervical segment of internal carotid artery; C2, petrous segment of internal carotid artery; C4, cavernous segment of internal carotid artery; *p < 0.05; **p < 0.01; ***p < 0.001.

### 3.2 Intergroup analysis of hemodynamic measurement and NVC measurement

For NVC measurement, this indicator reduced significantly, whether limited to GM or through the global cerebrum in CSVD compared to HC (*T* = 7.97, *p* < 0.01; *T* = 9.53, *p* < 0.01).

**Figure 3:**
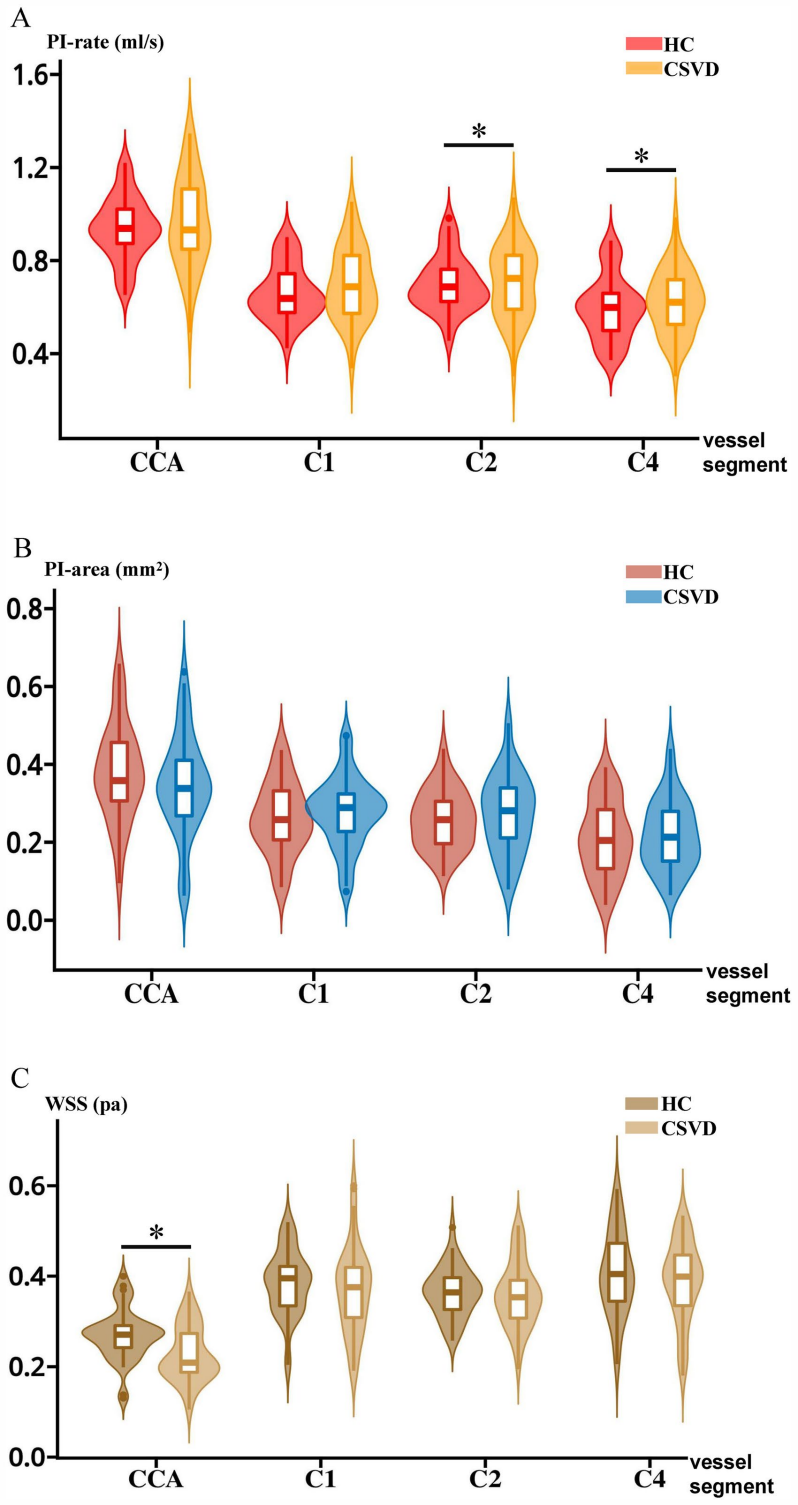
Intergroup comparisons of PI-rate, PI-area, and WSS between HC and CSVD. **(A)** Intergroup comparison between HC and CSVD in PI-rate. **(B)** Intergroup comparison between HC and CSVD in the PI-area. **(C)** Intergroup comparison between HC and CSVD in WSS. CCA, common carotid artery; C1, cervical segment of internal carotid artery; C2, petrous segment of internal carotid artery; C4, cavernous segment of internal carotid artery; HC, healthy control. CSVD, cerebral small vessel disease; PI, pulsatility index; WSS, wall shear stress.

For hemodynamic measurements, PI-rate-C2 and PI-rate-C4 increased significantly after age adjustment in CSVD compared to HC (*F* = 7.66, *p* < 0.01; *F* = 5.30, *p* = 0.02, respectively). Although there were no significant differences between HC and CSVD in PI across other vessel segments, a trend toward increased PI was observed in CSVD in PI-rate-CCA, PI-rate-C1, PI-area-C1, PI-area-C2, and PI-area-C4 (all *p* > 0.05) (except for PI-area-CCA). On the other hand, WSS-CCA decreased significantly after age adjustment in the CSVD group compared to that in the HC group (*F* = 15.75, *p* < 0.01). Although there were no significant differences in WSS between HC and CSVD in other vessel segments, a trend toward decreased WSS was generally observed in CSVD in WSS-C1, WSS-C2, and WSS-C4 (all *p* > 0.05). The above results are shown in Figure 3.

For additional hemodynamic measurements positively or negatively related to WSS, the mean cross-sectional area in CCA increased significantly after age adjustment in CSVD compared to HC (*F* = 8.00, *p* < 0.01). However, no significant difference was observed in the mean flow rate in CCA after age adjustment (Supplementary Table 1).

### 3.3 Multiple linear regression between hemodynamic measurement and NVC measurement in CSVD patients

NVC measurement was assessed as a dependent variable in the global cerebrum. In Model 1, significant correlations were observed between PI-rate-CCA and NVC measurements (*β* = −0.32, *p* = 0.02), as well as between WSS-C4 and NVC measurements (*β* = −0.39, *p* < 0.01). In Model 2, significant correlations were observed between WSS-C1 (*β* = 0.25, *p* = 0.03) and WSS-C4 (*β* = 0.29, *p* = 0.02) and NVC measurements. However, no significant correlation was observed in PI-rate-CCA after adjustment. In Model 3, significant correlations were observed between WSS-C1 (*β* = 0.25, *p* = 0.03) and PI-area-C4 (*β* = 0.28, *p* = 0.02) and NVC measurements. The above results are shown in Table 2.

**Table 2:** Multiple regression of PI, WSS, and NVC measurement in CSVD.

	Model 1	Model 2	Model 3
	*β*.std. (*p*)	*β*.std. (*p*)	*β*.std. (*p*)
PI-rate-CCA	−0.32 (0.020)*	−0.18 (0.170)	−0.08 (0.610)
PI-rate-C1	−0.27 (0.050)	−0.01 (0.970)	0.10 (0.490)
PI-rate-C2	−0.23 (0.100)	−0.05 (0.760)	−0.08 (0.550)
PI-rate-C4	−0.13 (0.380)	0.21 (0.200)	0.14 (0.330)
PI-area-CCA	0.05 (0.710)	0.02 (0.840)	0.09 (0.410)
PI-area-C1	0.06 (0.680)	0.21 (0.090)	0.17 (0.110)
PI-area-C2	−0.19 (0.180)	0.04 (0.780)	0.02 (0.840)
PI-area-C4	0.04 (0.810)	0.25 (0.060)	0.28 (0.020)*
WSS-CCA	0.11 (0.450)	0.14 (0.250)	0.02 (0.830)
WSS-C1	0.23 (0.100)	0.25 (0.030)*	0.26 (0.020)*
WSS-C2	0.04 (0.780)	<0.01 (0.980)	0.07 (0.50)
WSS-C4	0.39 (0.004)**	0.29 (0.02)*	0.11 (0.360)

Model 1: univariate regression model.

Model 2: multivariable regression model, controlled for age, sex, and education.

Model 3: multivariable regression model, controlled for age, sex, education, VRF-total-score, CS-PVS, BG-PVS, CMB, LI, and WMH Fazekas score.

PI, pulsatile index; WSS, wall shear stress; CCA, common carotid artery; C1, cervical segment of internal carotid artery; C2, petrous segment of internal carotid artery; C4, cavernous segment of internal carotid artery; CSVD, cerebral small vessel disease; VRF, vessel risk factor; CS, centrum semiovale; PVS, perivascular space; BG, basal ganglia; CMB, cerebral microbleed; WMH, white matter hyperintensity; LI, lacunar infarction.

*p < 0.05;

**p < 0.01;

***p < 0.001.

Additionally, multiple linear regression analysis was performed, whether limited to GM in the total CSVD cohort or global cerebrum in the subgroup CSVD cohort. Subgroup analyses were limited to the mild CSVD cohort (Rothwell criteria: score 1–2). (Supplementary Tables 2, 3). Moreover, similar results were observed when multiple linear regression was performed between hemodynamic and NVC measurements derived from the ReHo, which validated the robustness of this significant correlation derived from the ALFF (Supplementary Tables 4, 5).

### 3.4 Multiple linear regression between hemodynamic measurement and cognition scale in CSVD patients

When MMSE and MoCA scores were the dependent variables, no significant correlations between PI-area, PI-rate, WSS, and cognitive scores were observed across all vessel segments in the models (Supplementary Tables 6, 7).

### 3.5 Ordinal logistic regression of hemodynamic measurement and CSVD-total-burden-score in CSVD patients

CSVD-total-burden-score served as a dependent variable using Rothwell’s criteria. In Model 1, significant correlations were observed between WSS-C4 and CSVD-total-burden-score (OR < 0.01, 95%CI: <0.01/0.81). In Model 2, significant correlations were observed between WSS-C1 (OR < 0.01, 95%CI: <0.01/0.12), WSS-C2 (OR < 0.01, 95%CI: <0.01/0.5), and PI-rate-C4 (OR < 0.01, 95%CI: <0.01/0.08) and the CSVD-total-burden-score. However, no significant survival was observed in the WWS-C4 group after adjustment. The above results are shown in Supplementary Table 8.

CSVD-total-burden-score served as a dependent variable using Wardlaw’s criteria. In Model 1, significant correlations were observed between WSS-C2 (OR < 0.01, 95%CI: <0.01/0.6) and WSS-C4 (OR < 0.01, 95%CI: <0.01/0.41) and the CSVD-total-burden-score. In Model 2, significant correlations were observed between WSS-C1 (OR < 0.01, 95%CI: <0.01/0.13), WSS-C2 (OR < 0.01, 95%CI: <0.01/0.09), PI-rate-C4 (OR < 0.01, 95%CI: <0.01/0.28), and CSVD-total-burden-score. However, no significant survival was observed in the WSS-C4 group after adjustment. The above results are shown in Supplementary Table 9.

## 4 Discussion

In this study, interactions between hemodynamics and NVC were observed in patients with CSVD. The key findings were as follows: (1) Elevated PI-rate-C2, PI-rate-C4, and decreased WSS-CCA were observed in patients with CSVD compared to HC; (2) increased PI-area-C4 correlated with higher NVC measurement after adjustment, suggesting a dual effect of PI on brain functioning; and (3) WSS-C1 was negatively linked to NVC measurement after adjustment, implying a potential pathophysiological pathway between decreased WSS and neurovascular decoupling. Collectively, the WSS and PI could underscore their complex effects on NVC in CSVD pathophysiology.

### 4.1 Elevated PI-rate-C2 and PI-rate-C4 correlated with the presence of CSVD

According to previous research, PI is prevalently higher in multiple vessel segments in patients with CSVD (Shi et al., 2018; van Tuijl et al., 2022) and AD patients (Rivera-Rivera et al., 2016), significantly increased PI-rate-C2 and PI-rate-C4 in our study were observed in CSVD patients compared to HC, which may be partially due to the significantly higher hypertension rate in CSVD patients (Zieman et al., 2005). Besides, although there were no significant differences in PI between HC and CSVD in other vessel segments, a trend toward increased PI-rate and PI-area in CSVD patients was observed, suggesting that increased PI may be of varying sensitivity in alteration across vessel segments in early-stage CSVD, potentially reflecting regional changes in hemodynamics before overt clinical symptoms appear. These findings emphasize the importance of monitoring PI as a potential marker for early-stage CSVD, even in the absence of significant changes across all vessel segments.

### 4.2 Reduced WSS-CCA correlated with the presence of CSVD

Consistent with previous studies demonstrating a decreased WSS in CCA related to CSVD (Liu et al., 2016; Jeong and Rosenson, 2013), our study revealed a significantly lower WSS-CCA in CSVD patients. The WSS was positively proportional to flow rate and negatively proportional to vascular diameter (Cunningham and Gotlieb, 2005), with our study showing non-significant increases in flow rate and significant increases in vascular diameter. The latter is likely the primary factor contributing to the reduced WSS-CCA. In addition, a previous study has demonstrated that male sex, body height and weight, systolic blood pressure, and alcohol consumption were positively related to lumen diameter, while low-density lipoprotein cholesterol was negatively related (Bonithon-Kopp et al., 1996). In our study, a higher ratio of hypertension, male sex, alcohol consumption, and hyperlipoidemia in CSVD patients compared to HC were all in alignment with these findings and synergistically contributed to decreased WSS across vessel segments. Although only WSS-CCA reached statistical significance, a downward trend in WSS was observed across vessel segments in CSVD compared to HC. This trend may be interpreted by arterial tortuosity, characterized by abnormal twists and turns of one or several arteries and associated with older age, female sex, higher blood pressure, and cardiovascular risk factors (Ciurică et al., 2019). This condition possibly increases incidences of curvatures in CSVD patients, which further leads to disruption of lamina flow and consequently results in decreased WSS (Cunningham and Gotlieb, 2005).

### 4.3 Interactions between increased PI-area-C4 and NVC in mild CSVD

In contrast to previous studies demonstrating that higher PI correlates with increased WMH and brain atrophy (Jochemsen et al., 2015; Pahlavian et al., 2021; Webb et al., 2012; Shi et al., 2018), our study found that PI-area-C4 was positively correlated with NVC measurement, suggesting that higher PI contributes to sustaining regular brain activity rather than disruption. This correlation may be explained as follows: (1) elevated WMH and PVS preceded increased PI in cerebral arterial, elevated PI is a relatively late manifestation rather than a risk factor (Vikner et al., 2022), suggesting a complementary effect from structural disruption to dysfunction, and consequently to increased PI; (2) cerebral arterial pulsatility drove perivascular cerebral spinal fluid influx into, through the brain parenchyma, and drainage of toxic solutes (Iliff et al., 2013), implying a contribution to perivascular fluid movement and further normal brain function; (3) negative correlation between CSVD-total-burden and PI in multiple vessel segments, suggests a link of higher PI and structural integrity, and further NVC normality; and (4) vessel wall in CSVD patients is characterized by increased stiffness due to wall thickening, arteriolosclerosis (Wardlaw et al., 2013), and loss of smooth muscle cells (Hill and Meininger, 2012), making it harder to transfer sufficient blood to sustain regular activity in distal neurons and gliocytes, thus requiring increased blood supply with higher kinetic energy.

Additionally, similar results from a subgroup analysis further proved this correlation when we limited subjects to mild CSVD participants (Supplementary Tables 2, 3), implying a dynamic correlation across the progression of CSVD, which may lead to an insignificant correlation in early-stage CSVD but a significant correlation in more critical CSVD patients. In conclusion, we may refer to the fact that the positive effect of sustaining blood supply in microvascular circulation prevails over the negative impact of disruption on vessel wall due to increased PI in mild CSVD patients. This net effect consequently leads to a significant positive correlation between PI and NVC measurements even after confounders’ adjustment. However, it is difficult to extrapolate the results from our study population to patients with a more severe CSVD-total-burden. Heterogeneity, including vessel segments and cohorts across various research, may also contribute to this correlation.

### 4.4 WSS-C1 served as a negative predictor in CSVD

Decreased WSS-C1 was associated with lower NVC measurements in CSVD, indicating that lower WSS may promote neurovascular uncoupling. This correlation may be explained by two main mechanisms: (1) Combining particular systematic risk factors (hypertension, smoking, hypercholesterolemia, and diabetes mellitus), decreased WSS exerts an influence on cellular morphology and functions, which synergistically promotes atherosclerotic plaque formation (Cunningham and Gotlieb, 2005). Atherogenesis further narrows vascular diameter, elevates vascular resistance, results in insufficient blood supply in distal microcirculation, and ultimately impairs NVC; (2) the observed negative correlation between CSVD-total-burden and WSS in multiple vessel segments suggests that lower WSS exacerbates brain structural damage that could contribute to NVC dysfunction (Yang and Webb, 2023), which is consistent with another study demonstrating that decreased WSS in the CCA was correlated with increased WMH and reduced global cognitive performance (Liu et al., 2016). In conclusion, lower WSS may act as an independent risk factor in NVC dysfunction in CSVD patients.

### 4.5 Alterations of cognition performance

In contrast to research demonstrating that both PI and WSS are correlated with cognitive performance (Mitchell et al., 2011; Birnefeld et al., 2020; Pahlavian et al., 2021; Liu et al., 2016), no significant correlations were found between cognitive scores and hemodynamic measurements in patients with CSVD in our study. This lack of correlation may be because (1) the majority of participants were too mild in structural disruption to induce significant clinical alteration, (2) vessel segments concentrated on the carotid artery rather than the intracranial artery, and (3) there was heterogeneity of cohort and disease type, all of which suggest that alterations of hemodynamics in the carotid artery may be of low sensitivity in detecting cognitive performance in early-stage CSVD. Additionally, combining significant differences in NVC measurement and the insignificant difference in MoCA and MMSE scores between the CSVD and HC groups further supports the notion that NVC dysfunction in CSVD patients may precede noticeable global cognitive decline. On the other hand, the discussions that NVC measurement was reduced in CSVD compared to HC and the effects of hemodynamic measurements on NVC measurement were of heterogeneity, which are included in the Supplementary material.

### 4.6 Limitations

Limitations in our study were as follows: (1) The existence and functioning of a potential compensatory mechanism for increased PI in mild CSVD patients requires further investigation across various degrees of disease severity, with larger and longitudinal cohorts. (2) Although global cognitive scales were evaluated, it was suggested that more detailed and various scales are required in CSVD patients who are likely to suffer from multiple cognitive domains. (3) Structural disruption of the blood–brain barrier and neurovascular functioning still lacked demonstration in a large cohort of human brains, even though proved in a few human brains (Vikner et al., 2024); (4) NVC measurement in different brain regions may be of different sensitivities to hemodynamic measurements in the carotid artery, and further study may be beneficial from focusing on more specific brain areas rather than the global cerebrum; and (5) higher spatial resolution may improve accuracy in hemodynamic measurements of 4D flow MRI.

## 5 Conclusion

This study delineates the essential roles of PI and WSS in the pathophysiology of CSVD, where elevated PI and WSS contribute to sustaining normal neurovascular coupling. These findings highlight those hemodynamic measurements of the carotid arteries could serve as early indicators of CSVD.

## Data Availability

The raw data supporting the conclusions of this article will be made available by the authors without undue reservation.

## Footnotes

^1^ https://www.circlecvi.com/
