# Supplementary material for: Association between carotid artery hemodynamics and neurovascular coupling in cerebral small vessel disease: an exploratory study
Source: Front Aging Neurosci. 2025 Feb 7;17:1536552. doi: 10.3389/fnagi.2025.1536552 (PMC11842443; doi:10.3389/fnagi.2025.1536552)
Supplement: Supplementary file 1 [file Data_Sheet_1.docx]

**Supplementary material**

**2. Material and methods**

**2.5 MRI acquisition**

During the acquisition, the individuals in the supine position wore headphones while keeping their eyes closed without moving and awake without thinking about things. All participants were also instructed to relax as much as possible and to keep breathing steadily. Detailed acquisition parameters were as follows. (1) BOLD was acquired using a gradient recalled echo echo-planar imaging sequence with the following parameters: repetition time (TR) 2000.0ms, echo time (TE) 35.0 ms, flip angle 90 °, slice thickness 3.0 mm spacing 1.0 mm, field of view (FOV) 240.0 × 240.0 mm^2^, matrix size 80 × 80, reconstructed voxel size 3.0 × 3.0× 4.0 mm^3^, number of time points 128, each volume included 33 continuous slices covering the whole brain. Acquisition time was 4 minutes and 16 seconds. (2) Multi-delay pCASL sequence with the following parameters: TR 7618.0 ms, TE 11.3 ms, flip angle 111.0 °, slice thickness 4.0 mm without gap, FOV 240.0 × 240.0 mm^2^, reconstructed matrix size 128 × 128, labeling duration 3500.0 ms, the first post-labeling delay 1000.0 ms, the second post-labeling delay 2166.7 ms, the third post-labeling delay 3333.3 ms, number of excitations 1, number of slices 36, 6 arms with 600 points per arm, total scan time 4 minutes and 51 seconds with background suppression, and units: ml/100g/min. (3) High-resolution T1-weighted images were also obtained using magnetization-prepared rapid acquisition with gradient echo (MPRAGE) with the following parameters: TR 2713. 6 ms, TE 4.0 ms, inversion time 1000.0 ms, flip angle 8°, slice thickness 1.0 mm, no gaps, FOV 240.0 × 240.0 mm^2^, matrix size 256 × 256, reconstructed voxel size 1.0 × 1.0 × 1.0 mm^3^, number of slices 188, and total scan time 3 minutes and 29 seconds. (4) magnetic resonance image compilation (MAGiC) mapping was also obtained using MAGiC pulse sequence with the following parameters: TR 40000.0 ms, TE 94.1 ms, inversion time 11.1 ms, flip angle 90°, slice thickness 5.0 mm, 1.5 mm gaps, FOV 240.0 × 192.0 mm^2^, matrix size 256 × 256, reconstructed voxel size 0.5 × 0.5 × 6.5 mm^3^, number of slices 20, and total scan time 4 minutes. (5) Susceptibility-weighted imaging was also obtained using SWAN with the following parameters: TR 38.3 ms, TE 23.3 ms, flip angle 15°, slice thickness 2.0 mm, 1.0 mm gaps, FOV 240.0 × 240.0 mm^2^, matrix size 512 × 512, reconstructed voxel size 0.5 × 0.5 × 1 mm^3^, number of slices 128, and total scan time 3 minutes 25 seconds. (6) 4D flow data were acquired by 4D phase-contrast (PC) MRI, parameters of which: TR 4.34 ms, TE 2.42 ms, flip angle 8°, slice thickness 2.0 mm, no gaps, FOV 360 × 324 mm^2^, matrix size192×192, reconstructed voxel size 1.41 × 1.41 × 2 mm^3^, number of slices 82, velocity encoding 80 cm/s, bandwidth 651 Hz/pixel, sagittal orientation and total scan time 8 minutes. During the exam, a pulse oximeter was put onto the subject’s finger to collect the cardiac trigger and reconstruct images retrospectively. After acquisition, magnitude data providing the anatomic information and flow images representing the velocities of x-axis, y-axis, and z-axis were reconstructed into 20 frames per cardiac cycle.

**2.6 CSVD-total-burden-score evaluation**

Neuroimaging markers of CSVD, including WMH, LI, CMBs, and EPVS, were assessed based on neuroimaging evaluation by two bridle-wise neurologists (P Zeng, BN Li), with each neurologist being responsible for all four features blinded to the participants’ clinical information and each judgment. When inconsistent results were obtained, another senior neurologist (D Luo) who knew nothing about the original evaluations decided on the final assessment. In brief, WMH was estimated according to the Fazekas scale including deep white matter hyperintensities (DWMH) and periventricular white matter hyperintensities (PWMH) (total score: 0–6); the number of LI and CMBs were counted respectively; EPVS located in basal ganglia (BG-EPVS) and centrum semiovale (CS-EPVS), was respectively estimated using a previously validated semi-quantitative scale (grade: 0–4). The intraclass correlation coefficients (ICC) of CSVD characteristics between neurologists were as follows: DWMH, 0.819 (95%CI: 0.729-0.88); PWMH, 0.893 (95%CI: 0.843-0.928); CS-PVS, 0.883 (95%CI: 0.829-0.921); BG-EPVS, 0.75 (95%CI: 0.64-0.829); CMBs, 0.994 (95%CI: 0.992-0.996); LI, 0.886 (95%CI: 0.832-0.923), test-retest reliabilities of which were moderate to good, good to excellent, good to excellent, moderate to good, excellent to excellent, good to excellent, respectively.

All patients were classified into seven classes reflecting severity of CSVD overall burden according to Rothwell et al. criteria (score: 0-6).^1^ The specific standards were as follows: one point was assigned for total WMH with total Fazekas score 3–4, CMBs (numbers: 1-4), presence of lacunes, moderate-to-severe BG-EPVS or CS-EPVS (grade≥2), while two points were allocated for total WMH with total Fazekas score 5–6, CMBs (number≥5). Score zero represented condition without CSVD, while score 1-6 represented patients with increasing severity of CSVD. Wardlaw’s scale (scored: 0-4) criteria were also performed.^2^

**2.7 NVC measurements from BOLD and ASL**

BOLD data were preprocessed by an improved toolkit for resting-state functional magnetic resonance imaging data processing (RESTplus, version1.27, http://www.restfmri.net/forum/restplus) based on Statistical Parametric Mapping (SPM, Version 12. 0, <https://www.fil.ion.ucl.ac.uk/spm/software/spm12>) running on MATLAB (VersionR2018a, MathWorks, Natick, MA, USA). The procedures for each subject were as follows: (1) removing the first ten time points to ensure magnetization equilibrium; (2) slice timing of the remaining 118 volumes to correct time differences; (3) realignment to correct head motion and exclude individuals with displacement >3.0 mm or rotation >3.0 °; (4) spatial normalization of the functional images based on T1 structural images transformation (coregistration and segmentation) to non-linearly coregister to Montreal Neurological Institute (MNI) space at a resampling of 3.0 × 3.0 × 3.0 mm^3^; (5) linearly detrending of time series to remove signal drifting; (6) regression of nuisance covariates (Friston-24 motion parameters, white matter signals, cerebrospinal fluid signals), and (7) filtering to extract signal frequency between 0.01-0.08. (8) z-scored by subtracting the mean value and then dividing by the standard deviation (SD) within gray matter (GM) and global brain (remove cerebellum due to incomplete acquisition under current scanning scale), respectively. (9) smoothed by a Gaussian kernel with fullwidth at half-maximum (FWHM) of 6 mm.

Individual arterial-transit-time corrected CBF maps were acquired using FuncTool software (Version 4.7, GE Medical Systems, USA) based on a general kinetic model for ASL, then preprocessed using SPM 12 running in MATLAB R2018a and Advanced Normalization Tools (ANTS, http://stnava.github.io/ANTs) based on Linux. The detailed steps were as follows: (1) spatially normalizing the CBF maps to non-linearly coregister to standard ASL template in MNI space at a resampling of 3.0 × 3.0 × 3.0 mm^3^; (2) standardizing CBF map by z-scored within GM mask and removing non-brain tissues from each coregistered CBF map; (3) and smoothing by a Gaussian kernel with FWHM of 6 mm.

The regional homogeneity (ReHo) from BOLD is another indicator commonly used to describe the similarity of a given voxel with its nearest neighbors in terms of time series,^3^ representing regional homogeneity of neural activity and mapping local functional connectivity.^4^ The steps for calculating the ReHo from BOLD were as follows: (1) For each individual, Kendall’s coefficient concordance (KCC) was calculated according to its 26 neighboring voxels within a whole brain mask. (2) z-scored by subtracting the global mean value and then dividing by the SD within GM and global brain (remove cerebellum due to incomplete acquisition under current scanning scale), respectively. (3) smoothed by a Gaussian kernel with FWHM of 6 mm.

**4. Discussion**

**4.5 Alterations of cognition performance**

Significant positive cross-voxel correlations were observed in HC and CSVD respectively, implicating that neuronal activity and blood supply are coupled and thus supporting the theory that level of regional CBF follows intensity of neuronal activation.^7^ ^8^ Besides, significantly reduced cross-voxel correlation coefficients were found in CSVD compared to HC whether through the global cerebrum or limited in GM. The neurovascular unit, consisting of neurons, glial cells, and vascular components, underlays as structural basis of NVC. One previous study has demonstrated that CSVD-related hallmarks contribute to disruption of neurovascular units and further to neurovascular decoupling.^9^ Furthermore, astrocytes' damage in neurovascular unit may as well explain NVC dysfunction in CSVD to a certain extent.^10^

On other hand, PI-area, PI-rate, and WSS showed different significance to NVC measurement in multiple linear regression. Besides, even in the same dynamic measurement, all vessel segments showed different significance to NVC measurement. It is reasonable to refer to the fact that not only distinctive flow measurements, but also various locations of vessel segments proved different sensitivity to NVC measurement, providing potential explanations of mechanisms in terms of blood dynamic pathophysiology in CSVD.

| **Supplementary Table 1**. The intergroup difference in mean area and mean rate in CCA adjusted for age. | | | | |
| --- | --- | --- | --- | --- |
|  | HC *(*n=41) | CSVD *(*n=52) | F | ρ |
| mean-rate-CCA | 4.88 *(*1.01) | 5.13 *(*1.20) | 3.55 | 0.06 |
| mean-area-CCA | 31.94 *(*6.55) | 36.16 *(*7.50) | 8.00 | 0.006** |
| Variables were presented as mean (SD). HC: healthy control; CSVD: cerebral small vessel disease; PI: pulsatile index; CCA: common carotid artery; *: p<0.05. **: p<0.01. ***: p<0.001. | | | | |

| **Supplementary Table 2.** Multiple linear regression of PI, WSS, and cross-voxel-correlation-coefficient limited in global cerebrum in mild CSVD cohort. | |
| --- | --- |
|  | β.std (p) |
| PI-rate-CCA | 0.04 (0.77) |
| PI-rate-C1 | -0.01 (0.95) |
| PI-rate-C2 | -0.21 (0.26) |
| PI-rate-C4 | 0.22 (0.33) |
| PI-area-CCA | 0.04 (0.77) |
| PI-area-C1 | 0.13 (0.39) |
| PI-area-C2 | -0.12 (0.46) |
| PI-area-C4 | 0.31 (0.05) * |
| WSS-CCA | 0 (0.99) |
| WSS-C1 | 0.34 (0.02) * |
| WSS-C2 | 0.07 (0.62) |
| WSS-C4 | 0.11 (0.49) |
| Analysis was controlled for age, sex, education, VRF total score, CS-PVS, BG-PVS, CMB, LI, and WMH Fazekas score. PI: pulsatile index; WSS: wall shear stress; CCA: common carotid artery; C1: cervical segment of internal carotid artery; C2: petrous segment of internal carotid artery; C4: cavernous segment of internal carotid artery; CSVD: cerebral small vessel disease. VRF: vessel risk factor. CS: centrum semiovale. PVS: perivascular space. BG: basal ganglia. CMB: cerebral microbleed. WMH: white matter hyperintensity. LI: lacunar infarction. *: p<0.05. **: p<0.01. ***: p<0.001 | |

| **Supplementary Table 3**. Multiple linear regression of PI, WSS, and cross-voxel-correlation-coefficient limited in GM in total CSVD cohort. | |
| --- | --- |
|  | β.std (p) |
| PI-rate-CCA | -0.11 (0.44) |
| PI-rate-C1 | 0.04 (0.82) |
| PI-rate-C2 | -0.13 (0.4) |
| PI-rate-C4 | 0.08 (0.65) |
| PI-area-CCA | 0.15 (0.23) |
| PI-area-C1 | 0.06 (0.65) |
| PI-area-C2 | -0.03 (0.83) |
| PI-area-C4 | 0.27 (0.06) |
| WSS-CCA | 0.1 (0.42) |
| WSS-C1 | 0.3 (0.03) * |
| WSS-C2 | 0.19 (0.11) |
| WSS-C4 | 0.12 (0.37) |
| Analysis was controlled for age, sex, education, VRF total score, CS-PVS, BG-PVS, CMB, LI, and WMH Fazekas score. PI: pulsatile index; WSS: wall shear stress; CCA: common carotid artery; C1: cervical segment of internal carotid artery; C2: petrous segment of internal carotid artery; C4: cavernous segment of internal carotid artery; CSVD: cerebral small vessel disease. GM: grey matter. *: p<0.05. **: p<0.01. ***: p<0.001 | |

| **Supplementary Table 4**. Multiple linear regression of PI, WSS, and cross-voxel-correlation-coefficient using ReHo in total CSVD. | | | |
| --- | --- | --- | --- |
|  | model 1 | model 2 | model 3 |
|  | β.std (p) | β.std (p) | β.std (p) |
| PI-rate-CCA | -0.13 (0.38) | -0.03 (0.82) | 0.06 (0.67) |
| PI-rate-C1 | -0.09 (0.51) | 0.13 (0.47) | 0.11 (0.54) |
| PI-rate-C2 | -0.05 (0.73) | 0.07 (0.65) | 0.07 (0.67) |
| PI-rate-C4 | 0.01 (0.92) | 0.24 (0.18) | 0.26 (0.15) |
| PI-area-CCA | 0.06 (0.67) | <0.01 (0.99) | 0.05 (0.69) |
| PI-area-C1 | 0.1 (0.47) | 0.18 (0.17) | 0.12 (0.38) |
| PI-area-C2 | -0.07 (0.65) | 0.10 (0.48) | 0.04 (0.81) |
| PI-area-C4 | 0.1 (0.49) | 0.22 (0.12) | 0.18 (0.22) |
| WSS-CCA | 0.01 (0.93) | 0.03 (0.79) | -0.05 (0.71) |
| WSS-C1 | 0.18 (0.20) | 0.21 (0.09) | 0.30 (0.03) * |
| WSS-C2 | 0.16 (0.25) | 0.13 (0.31) | 0.18 (0.16) |
| WSS-C4 | 0.29 (0.04) * | 0.17 (0.18) | 0.05 (0.71) |
| Model 1: univariate regression model.  Model 2: multivariable regression model, controlled for age, sex, and education.  Model 3: multivariable regression model, controlled for age, sex, education, VRF total score, CS-PVS, BG-PVS, CMB, LI, and WMH Fazekas score. Abbreviations: PI: pulsatile index; WSS: wall shear stress; CCA: common carotid artery; C1: cervical segment of internal carotid artery; C2: petrous segment of internal carotid artery; C4: cavernous segment of internal carotid artery; CSVD: cerebral small vessel disease; VRF: vessel risk factor; CS: centrum semiovale; PVS: perivascular space; BG: basal ganglia; CMB: cerebral microbleed; WMH: white matter hyperintensity; LI: lacunar infarction.  *: p<0.05  **: p<0.01  ***: p<0.001 | | | |

| **Supplementary Table 5**. Multiple linear regression of PI, WSS, and cross-voxel-correlation-coefficient using ReHo in mild CSVD. | |
| --- | --- |
|  | β.std (p) |
| PI-rate-CCA | 0.08 (0.64) |
| PI-rate-C1 | 0.08 (0.70) |
| PI-rate-C2 | 0.01 (0.97) |
| PI-rate-C4 | 0.41 (0.09) |
| PI-area-CCA | 0.03 (0.87) |
| PI-area-C1 | 0.13 (0.41) |
| PI-area-C2 | 0.03 (0.88) |
| PI-area-C4 | 0.35 (0.04) * |
| WSS-CCA | <0.01 (0.99) |
| WSS-C1 | 0.35 (0.03) * |
| WSS-C2 | 0.14 (0.37) |
| WSS-C4 | 0.09 (0.61) |
| Model was controlled for age, sex, education, VRF total score, CS-PVS, BG-PVS, CMB, LI, and WMH Fazekas score. PI: pulsatile index; WSS: wall shear stress; CCA: common carotid artery; C1: cervical segment of internal carotid artery; C2: petrous segment of internal carotid artery; C4: cavernous segment of internal carotid artery; *: p<0.05. **: p<0.01. ***: p<0.001 | |

| **Supplementary Table 6.** Multiple linear regression of PI, WSS, and MoCA in CSVD. | | | |
| --- | --- | --- | --- |
|  | model 1 | model 2 | model 3 |
|  | β.std (p) | β.std (p) | β.std (p) |
| PI-rate-CCA | 0.03 (0.82) | 0.16 (0.20) | 0.27 (0.06) |
| PI-rate-C1 | 0.02 (0.89) | 0.17 (0.27) | 0.22 (0.19) |
| PI-rate-C2 | -0.09 (0.51) | 0.07 (0.64) | 0.09 (0.54) |
| PI-rate-C4 | -0.12 (0.39) | 0.1 (0.53) | 0.16 (0.36) |
| PI-area-CCA | -0.05 (0.74) | 0.11 (0.32) | 0.12 (0.33) |
| PI-area-C1 | 0.05 (0.73) | 0.18 (0.12) | 0.18 (0.16) |
| PI-area-C2 | -0.08 (0.58) | <0.01 (0.1) | -0.01 (0.92) |
| PI-area-C4 | -0.13 (0.37) | <0.01 (0.99) | -0.02 (0.88) |
| WSS-CCA | -0.07 (0.63) | -0.11 (0.31) | -0.14 (0.24) |
| WSS-C1 | 0.07 (0.61) | 0.06 (0.60) | 0.05 (0.77) |
| WSS-C2 | 0.13 (0.35) | 0.14 (0.22) | 0.11 (0.52) |
| WSS-C4 | 0.07 (0.61) | 0.05 (0.64) | 0.01 (0.95) |
| Model 1: univariate regression model.  Model 2: multivariable regression model, controlled for age, sex, and education.  Model 3: multivariable regression model, controlled for age, sex, education, VRF total score, CS-PVS, BG-PVS, CMB, LI, and WMH Fazekas score. Abbreviations: PI: pulsatile index; WSS: wall shear stress; MoCA: montreal cognition assessment. CCA: common carotid artery; C1: cervical segment of internal carotid artery; C2: petrous segment of internal carotid artery; C4: cavernous segment of internal carotid artery; CSVD: cerebral small vessel disease; VRF: vessel risk factor; CS: centrum semiovale; PVS: perivascular space; BG: basal ganglia; CMB: cerebral microbleed; WMH: white matter hyperintensity; LI: lacunar infarction. *: p<0.05  **: p<0.01  ***: p<0.001 | | | |

| **Supplementary Table 7.** Multiple linear regression of PI, WSS, and MMSE in CSVD. | | | |
| --- | --- | --- | --- |
|  | model 1 | model 2 | model 3 |
|  | β.std (p) | β.std (p) | β.std (p) |
| PI-rate-CCA | -0.10 (0.50) | 0.02 (0.87) | 0.06 (0.71) |
| PI-rate-C1 | 0.02 (-0.90) | 0.30 (0.10) | 0.33 (0.10) |
| PI-rate-C2 | -0.12 (0.40) | 0.08 (0.66) | 0.07 (0.71) |
| PI-rate-C4 | -0.11 (0.45) | 0.18 (0.34) | 0.23 (0.27) |
| PI-area-CCA | -0.07 (0.62) | 0.03 (0.83) | 0.08 (0.60) |
| PI-area-C1 | 0.07 (0.63) | 0.21 (0.14) | 0.22 (0.15) |
| PI-area-C2 | -0.01 (0.94) | 0.13 (0.40) | 0.07 (0.69) |
| PI-area-C4 | 0.04 (0.78) | 0.23 (0.12) | 0.28 (0.09) |
| WSS-CCA | -0.03 (0.81) | -0.05 (0.72) | -0.11 (0.45) |
| WSS-C1 | -0.011 (0.94) | -0.03 (0.82) | -0.02 (0.88) |
| WSS-C2 | 0.03 (0.83) | 0.03 (0.81) | 0.04 (0.78) |
| WSS-C4 | 0.17 (0.23) | 0.03 (0.81) | 0.17 (0.31) |
| Model 1: univariate regression model.  Model 2: multivariable regression model, controlled for age, sex, and education.  Model 3: multivariable regression model, controlled for age, sex, education, VRF total score, CS-PVS, BG-PVS, CMB, LI, and WMH Fazekas score. Abbreviations: PI: pulsatile index; WSS: wall shear stress; MMSE: mini-mental state evaluation. CCA: common carotid artery; C1: cervical segment of internal carotid artery; C2: petrous segment of internal carotid artery; C4: cavernous segment of internal carotid artery; CSVD: cerebral small vessel disease; VRF: vessel risk factor; CS: centrum semiovale; PVS: perivascular space; BG: basal ganglia; CMB: cerebral microbleed; WMH: white matter hyperintensity; LI: lacunar infarction. *: p<0.05  **: p<0.01  ***: p<0.001 | | | |

| **Supplementary Table 8.** Ordinal logistic regression of PI, WSS, and CSVD-total-burden-score using Rothwell’s criteria in CSVD. | | |
| --- | --- | --- |
|  | model 1 | model 2 |
|  | OR (95%CI） | OR (95%CI） |
| PI-rate-CCA | 8.20 (0.44/151.71) | 0.2 (<0.01/10.35) |
| PI-rate-C1 | 21.24 (0.56/803.52) | 0.33 (<0.01/102.82) |
| PI-rate-C2 | 3.46 (0.10/118.75) | 0.02 (<0.01/3.39) |
| PI-rate-C4 | 2.94 (0.05/160.93) | <0.01 (<0.01/0.08) * |
| PI-area-CCA | 0.75 (0.01/55.76) | 0.03 (<0.01/16.89) |
| PI-area-C1 | 9.17 (0.02/5135.85) | 3.15 (<0.01/21183.97) |
| PI-area-C2 | 5.08 (0.02/1327.43) | 0.09 (<0.01/168.85) |
| PI-area-C4 | 73.04 (0.13/39656.10) | 2.96 (<0.01/5658.99) |
| WSS-CCA | <0.01 (<0.01/33.15) | 0.03 (<0.01/966.81) |
| WSS-C1 | 0.01 (<0.01/5.4) | <0.01 (<0.01/0.12) * |
| WSS-C2 | <0.01 (<0.01/4.22) | <0.01 (<0.01/0.50) * |
| WSS-C4 | <0.01 (<0.01/0.81) * | 0.04 (<0.01/92.57) |
| Model 1: univariate regression model.  Model 2: multivariable regression model, controled for age, sex, education, and VRF total score. Abbreviations:PI: pulsatile index; WSS: wall shear stress; CCA: common carotid artery; C1: cervical segment of internal carotid artery; C2: petrous segment of internal carotid artery; C4: cavernous segment of internal carotid artery; CSVD: cerebral small vessel disaese; VRF: vessel risk factor;  * : p<0.05  ** : p<0.01  *** : p<0.001 | | |

s

| **Supplementary Table 9.** Ordinal logistic regression of PI，WSS and CSVD-total-burden-score using Wardlaw’s crateria in CSVD patients. | | |
| --- | --- | --- |
|  | model 1 | model 2 |
|  | OR (95%CI） | OR (95%CI） |
| PI-rate-CCA | 2.66 (0.17/40.81) | 0.13 (<0.01/4.00) |
| PI-rate-C1 | 2.6 (0.09/77.40) | 0.06 (<0.01/11.11) |
| PI-rate-C2 | 0.52 (0.02/15.17) | 0.01 (<0.01/1.17) |
| PI-rate-C4 | 0.53 (0.01/25.18) | <0.01 (<0.01/0.28) * |
| PI-area-CCA | 1.35 (0.02/84.86) | 0.36 (<0.01/40.69) |
| PI-area-C1 | 1.45 (<0.01/583.47) | 0.25 (<0.01/337.65) |
| PI-area-C2 | 0.50 (<0.01/105.64) | 0.01 (<0.01/6.79) |
| PI-area-C4 | 17.24 (0.04/7193.98) | 1.16 (<0.01/1456.80) |
| WSS-CCA | <0.01 (<0.01/43.08) | 0.05 (<0.01/670.48) |
| WSS-C1 | 0.01 (<0.01/3.87) | <0.01 (<0.01/0.13) * |
| WSS-C2 | <0.01 (<0.01/0.6) * | <0.01 (<0.01/0.09) * |
| WSS-C4 | <0.01 (<0.01/0.41) * | 0.03 (<0.01/30.63) |
| Model 1: univariate regression model.  Model 2: multivariable regression model, controled for age, sex, education, and VRF total score. Abbreviations:PI: pulsatile index; WSS: wall shear stress; CCA: common carotid artery; C1: cervical segment of internal carotid artery; C2: petrous segment of internal carotid artery; C4: cavernous segment of internal carotid artery; CSVD: cerebral small vessel disaese; VRF: vessel risk factor; CS: centrum semiovale;  * : p<0.05  ** : p<0.01  *** : p<0.001 | | |

**Reference**

1 Lau KK, Li L, Schulz U, Simoni M, Chan KH, Ho SL *et al.* Total small vessel disease score and risk of recurrent stroke: Validation in 2 large cohorts. *Neurology* 2017; **88**: 2260–2267.

2 Wardlaw JM, Smith EE, Biessels GJ, Cordonnier C, Fazekas F, Frayne R *et al.* Neuroimaging standards for research into small vessel disease and its contribution to ageing and neurodegeneration. *Lancet Neurol* 2013; **12**: 822–838.

3 Zang Y, Jiang T, Lu Y, He Y, Tian L. Regional homogeneity approach to fMRI data analysis. *Neuroimage* 2004; **22**: 394–400.

4 Jiang L, Zuo X-N. Regional Homogeneity: A Multimodal, Multiscale Neuroimaging Marker of the Human Connectome. *Neuroscientist* 2016; **22**: 486–505.

5 Xie L, Zhang Y, Hong H, Xu S, Cui L, Wang S *et al.* Higher intracranial arterial pulsatility is associated with presumed imaging markers of the glymphatic system: An explorative study. *Neuroimage* 2024; **288**: 120524.

6 Bouthillier A, van Loveren HR, Keller JT. Segments of the internal carotid artery: a new classification. *Neurosurgery* 1996; **38**: 425–432; discussion 432-433.

7 Venkat P, Chopp M, Chen J. New insights into coupling and uncoupling of cerebral blood flow and metabolism in the brain. *Croat Med J* 2016; **57**: 223–228.

8 Stobart JL, Anderson CM. Multifunctional role of astrocytes as gatekeepers of neuronal energy supply. *Front Cell Neurosci* 2013; **7**: 38.

9 Yang S, Webb AJS. Associations between neurovascular coupling and cerebral small vessel disease: A systematic review and meta-analysis. *European Stroke Journal* 2023; **8**: 895–903.

10 Stobart JL, Anderson CM. Multifunctional role of astrocytes as gatekeepers of neuronal energy supply. *Front Cell Neurosci* 2013; **7**: 38.
